# Strategy to improve synaptic behavior of ion-actuated synaptic transistors—the use of ion blocking layer to improve state retention

**DOI:** 10.1038/s41598-024-55681-7

**Published:** 2024-02-29

**Authors:** Seonuk Jeon, Nir Tessler, Nayeon Kim, Eunryeong Hong, Hyun Wook Kim, Jiyong Woo

**Affiliations:** 1 School of Electronic and Electrical Engineering, Kyungpook National University, Daegu, 41566 South Korea; 2 Electrical and Computer Engineering, Sara and Moshe Zisapel Nano-Electronic Center, Technion Israel Institute of Technology, 32000003 Haifa, Israel; 3 Department of Earth and Environmental Engineering, Columbia University, New York, NY 10027 USA

**Keywords:** Electrical and electronic engineering, Electronic devices

## Abstract

Synaptic transistors (STs) with a gate/electrolyte/channel stack, where mobile ions are electrically driven across the solid electrolyte, have been considered as analog weight elements for neuromorphic computing. The current (I_D_) between the source and drain in the ST is analogously updated by gate voltage (V_G_) pulses, enabling high pattern recognition accuracy in neuromorphic systems; however, the governing physical mechanisms of the ST are not fully understood yet. Our previous physics-based simulation study showed that ion movement in the electrolyte, rather than the electrochemical reactions that occur in the channel, plays an important role in switching. In this study, we experimentally explore the properties of the HfO_x_ electrolyte and show that by tuning the density of oxygen vacancies, it can assume the dual role of electrolyte and channel. We demonstrate analog synaptic behavior using a novel ST with a two-layer stack of CuO_x_/HfO_x_, where the CuO_x_ is the gate and Cu ion reservoir, and the HfO_x_ is the electrolyte and channel. To improve state retention and linearity, we introduce a Cu ion transport barrier in the form of a dense and stoichiometric Al_2_O_3_ layer. The CuO_x_/Al_2_O_3_/HfO_x_ exhibits excellent state retention and improved potentiation and depression response. Energy dispersive spectroscopy mapping following potentiation confirms the role of the Al_2_O_3_ layer in confining the Cu ions in the HfO_x_ layer. We also show that a two-step programming scheme can further enhance synaptic response and demonstrate high recognition accuracy on the Fashion-MNIST dataset in simulation.

## Introduction

Recently, with the rapid increase in the amount of data, the conventional von Neumann architecture, which processes data through a series of operations between the processing unit and memory, has created a bottleneck effect that slows data processing. To overcome this, neuromorphic computing architecture, based on highly parallel analog computations inspired by data transmission through numerous synapses in the human brain, has been attracting attention^1–3^. To implement this architecture in hardware, a synaptic device that emulates the role of a biological synapse is required^4,5^. Static random-access memory (SRAM) has been utilized as a synaptic device; however, owing to the large size of the SRAM cell (over 100 F^2^, where F is the technology node), it is challenging to implement hundreds of millions of synapses in neuromorphic computing systems^6^. For this reason, various two-terminal emerging memory devices such as ferroelectric memory^7^, magnetic memory^8^, phase change memory^9–11^, and resistive memory (RRAM)^12^ have been proposed. Among these, RRAM has been mainly explored owing to its low power consumption, sub-10 nm scaling, nonvolatility, and multilevel characteristics^13–15^. However, its filamentary switching mechanism inevitably leads to resistance states, indicating that the synaptic weights are probabilistically tuned, which causes performance degradation in pattern recognition applications^16^. This necessitates a new ion-actuated three-terminal synaptic transistor (ST) with a gate/electrolyte/channel stack for predictable and tunable analog synaptic weights^17^. The physical mechanism of the ST has not yet been elucidated; nevertheless, its plausible working principle has been mainly described by a two-step process: (*i*) ion migration through solid electrolyte and (*ii*) electrochemical reaction at the channel^18^. When a positive gate voltage (V_G_) pulse is applied to the gate of the ST, mobile ions originating from the gate or incorporated into the electrolyte are driven toward the channel in the vertical direction. Various mobile ions such as Li^+^^17,19^, O^2−^^20,21^, H^+^^22,23^, and Cu^+^^24,25^ have been explored. The broadly accepted picture is that the switching takes place only when the ions reach the channel and directly dope or convert the valence state of the channel’s atoms. Thus, the extent of the ions intercalated into the channel, which is related to the electrochemical potential applied to the gate stack by the V_G_ bias, analogously increases or decreases the current between the source and drain (I_D_), corresponding to potentiation or depression, respectively. We have recently shown that electrolyte charging also induces a charge in the channel material and that, in some cases, this may be the preferred mechanism^26^. Moreover, our physics-based simulation results of CuO_x_-gate/HfO_x_-electrolyte/WO_x_-channel stacks have shown that Cu intercalation into the WO_x_ channel leads to Cu plating, loss of linearity, and enhanced degradation^18^.

In this study, we focus on the HfO_x_ layer and use a CuO_x_/HfO_x_ ST to demonstrate that by tuning the density of oxygen vacancies, it can also assume the role of the conducting channel making the WO_x_ layer redundant. We also show that an ultrathin Al_2_O_3_ film, inserted between the CuO_x_ and HfO_x_ layers, acts as an ion barrier that confines the Cu ions to the HfO_x_ layer and improves the state retention and linearity.

**Figure 1:**
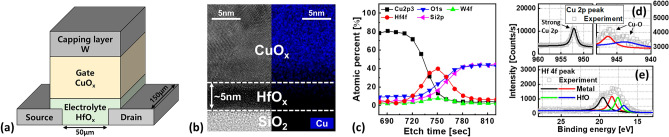
(**a**) Schematic diagram, (**b**) cross-sectional TEM image, and (**c**) XPS depth profiling of the fabricated CuO_x_/HfO_x_ ST. (**d**) Cu 2p and (**e**) Hf 4f peak intensities of CuO_x_ and HfO_x_ layers, respectively.

The three-terminal STs composed of all CMOS compatible electrodes and layers were formed on a Si substrate with a thermally grown 100 nm-thick SiO_2_ wafer. First, as shown in Fig. 1a, source (S) and drain (D) contacts were patterned and deposited by sputtering with W target at a power of 50 W. The patterning was performed via conventional photolithography, developing, and lift-off processes. Afterwards, a 5 nm-thick HfO_x_ electrolyte with a length (width) of 50 (150) μm was deposited by sputtering with HfO_2_ target at a power of 100 W under Ar and O_2_ gas flows at the rate of 25 and 5 sccm, respectively. Next, a 360 nm-thick CuO_x_ gate electrode was deposited by sputtering with Cu target at 100 W using Ar and O_2_ gases at rates of 27 and 3 sccm, respectively. Finally, a W capping layer was deposited to prevent unwanted copper oxidation. The fabricated CuO_x_/HfO_x_ ST was analyzed by transmission electron microscopy (TEM) and X-ray photoelectron spectroscopy (XPS), as shown in Fig. 1b,c. As mentioned in our recent publications^25^, to limit the number of mobile Cu ions participating in I_D_ switching, the CuO_x_ gate electrode was adopted instead of the previously proposed Cu gate. As shown in Fig. 1d, the intensity of the Cu–O bonding at binding energies of 943 and 948 eV was detected in the measured Cu 2p peak^27^. Further, a non-stoichiometric HfO_x_ layer comprising both Hf–Hf metal and Hf–O oxide bonds were observed (Fig. 1e)^28^. As discussed below, we consider the oxygen vacancies as facilitators of copper ions transport.

**Figure 2:**
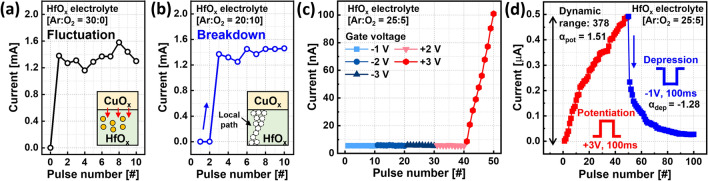
(**a**,**b**) Impact of Ar and O_2_ gas flow rates during HfO_x_ deposition on the I_D_ response of the CuO_x_/HfO_x_ ST. (**c**) I_D_ response of the CuO_x_/HfO_x_ ST, with optimized HfO_x_ stoichiometry, as a function of polarity and amplitude of V_G_. (**d**) The update curve of I_D_ in the ST employing optimized HfO_x_ electrolyte layer.

Figure 2 shows the importance of the HfO_x_ stoichiometry and the role of oxygen vacancies in determining the electrolyte’s properties. Figure 2a shows the response for a device using the previously developed HfO_x_^25^, sputtered using 30 sccm Ar only. Although a low initial I_D_ of 28 nA was obtained, the potentiation pulses of V_G_ = + 3 V and a pulse width of 100 ms flood the entire layer with Cu ions, thus reaching the maximum current within the first pulse. Applying the read voltage of 0.5 V to D and 0 V to S results in a current of about a 1.4 mA. Since the Cu ions transport is expected to be assisted by oxygen vacancies, we introduced oxygen flow and used sccm of 20:10 Ar to O_2_, respectively. The response to potentiation pulses is shown in Fig. 2b. Reduced oxygen vacancies render the HfO_x_ insulating to electron and Cu ion transport. Following a few pulses with low I_D_, the current abruptly jumps to its maximum value. It means that the high field concentrated across the insulating HfO_x_ layer led to permanent oxide breakdown (inset to Fig. 2b), resulting in low gate controllability.

The response of an optimized device using an HfO_x_ sputtered under 25:5 Ar:O_2_ flow is shown in Fig. 2c,d. Figure 2c, shows the response to potentiation by 10 pulses of 100 ms width and V_G_ values between − 3 V and 3 V. We note that V_G_ = 3 V initiates a linear potentiation response with the Cu ions transport into the 5 nm HfO_x_ layer being well controlled. An extended potentiation/depression response using 50 pulses is shown in Fig. 2d. While not being an ideal response, it clearly demonstrates the importance of controlling the density of oxygen vacancies to achieve well behaved gate controlled uniform Cu-ion migration throughout the electrolyte.

Based on the synaptic behavior in Fig. 2d, the linearity factor, α, was calculated by the following equation^29^:

$${\text{G}} = \left\{ {\begin{array}{*{20}c} {\left( {\left( {G_{MAX}^{{\upalpha }} - G_{MIN}^{{\upalpha }} } \right) \times \omega + G_{MIN}^{{\upalpha }} } \right)^{{\frac{1}{{\upalpha }}}} } & {\left( {if {\alpha } \ne 0} \right)} \\ {G_{MIN} \times \left( {G_{MAX} /G_{MIN} } \right)^{\omega } } & {\left( {if {\alpha } = 0} \right)} \\ \end{array} } \right.,$$

where, G_MAX_ and G_MIN_ are conductance at the maximum and minimum I_D_ state, respectively, and $\omega$ is an internal variable which ranges from 0 to 1. Moreover, α is equal to 1 in the case of the ideal synaptic behavior. Based on these equations, linearity of potentiation (α_pot_) of 1.51 was achieved during potentiation in CuO_x_/HfO_x_ ST. A hint for the process causing the sublinear potentiation can be found in the depression response to negative V_G_ pulses with amplitude of − 1 V and pulse width of 100 ms. The first pulse results in more than 50% reduction of the current with the response to the following pulses saturating quickly. The resulting a non-linear response has a linearity of depression (α_dep_) of − 1.28. Based on the results of Fig. 2d, we postulated that the nonlinearity is associated with facile Cu ions transport out of the HfO_x_ layer.

**Figure 3:**
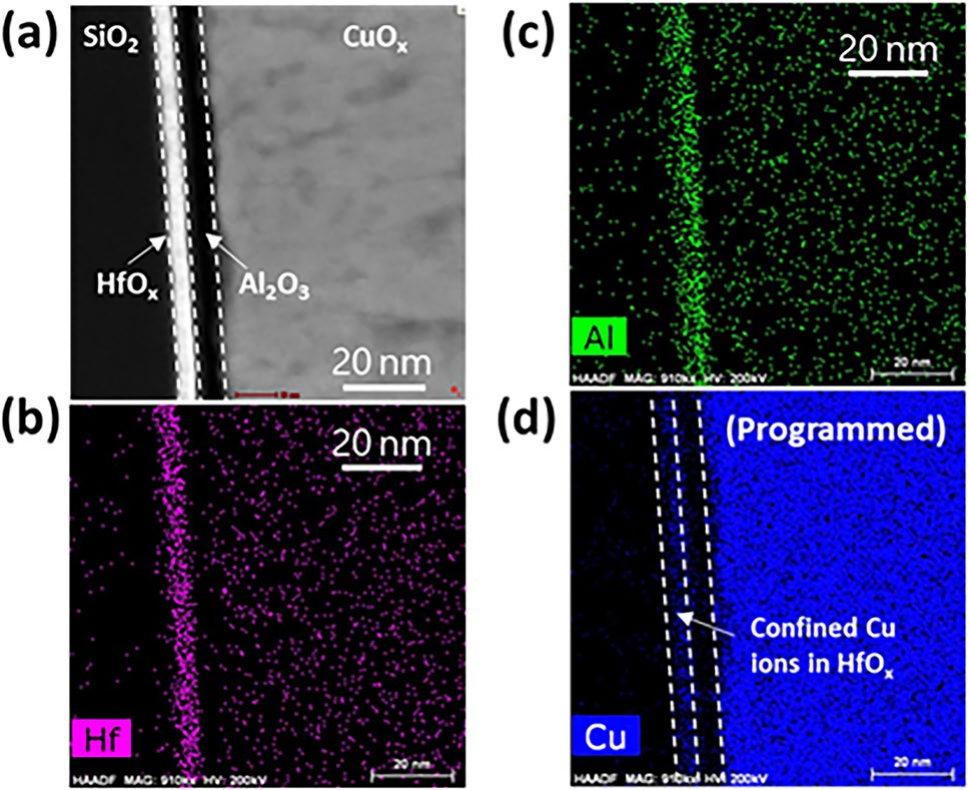
(**a**) HAADF-STEM image of the CuO_x_/Al_2_O_3_/HfO_x_ stack. (**b**,**c**) EDS mapping image showing the position of the HfO_x_ layer through (**b**) the Hf signal and of the Al_2_O_3_ layer through (**c**) the Al signal. (**d**) EDS mapping of CuO_x_/Al_2_O_3_/HfO_x_ ST following 100 potentiation pulses showing the Al_2_O_3_ layer being free of Cu ions which are being confined to the HfO_x_ layer.

To improve the retention of the Cu ions within the HfO_x_ layer we introduced a 2 nm Al_2_O_3_ film between CuO_x_ and HfO_x_. We used atomic layer deposition to ensure a relatively dense and stoichiometric layer that would serve as a partial barrier for Cu ion transport. The use of ultrathin Al_2_O_3_ film is important to avoid introducing extra ion resistance that may hamper the dynamic range. The deposition was done at a chamber temperature of 200 °C using trimethylaluminum and water sources. The approximately 2 nm-thick Al_2_O_3_ layer was deposited at a deposition rate of approximately 1.1 Å/cycle. Figure 3a shows an high-angle annular dark-field scanning transmission electron microscopy (HAADF-STEM) image of the CuO_x_/Al_2_O_3_/HfO_x_ stack where energy dispersive X-ray spectroscopy (EDS) mapping of Hf (Fig. 3b) and of Al (Fig. 3c) confirm the layers’ position. Figure 3d shows Cu mapping following 100 potentiation pulses of the CuO_x_/Al_2_O_3_/HfO_x_ ST. We note a uniform distribution within the HfO_x_ layer and no Cu signal within the Al_2_O_3_ one. The uniformity in the HfO_x_ layer is a testament to the successful morphology and stoichiometry supporting uniform Cu ion injection. Most importantly, the absence of Cu signal in the Al_2_O_3_ layer supports the notion that it acts as a barrier where the particles can only go through but not reside within.

**Figure 4:**
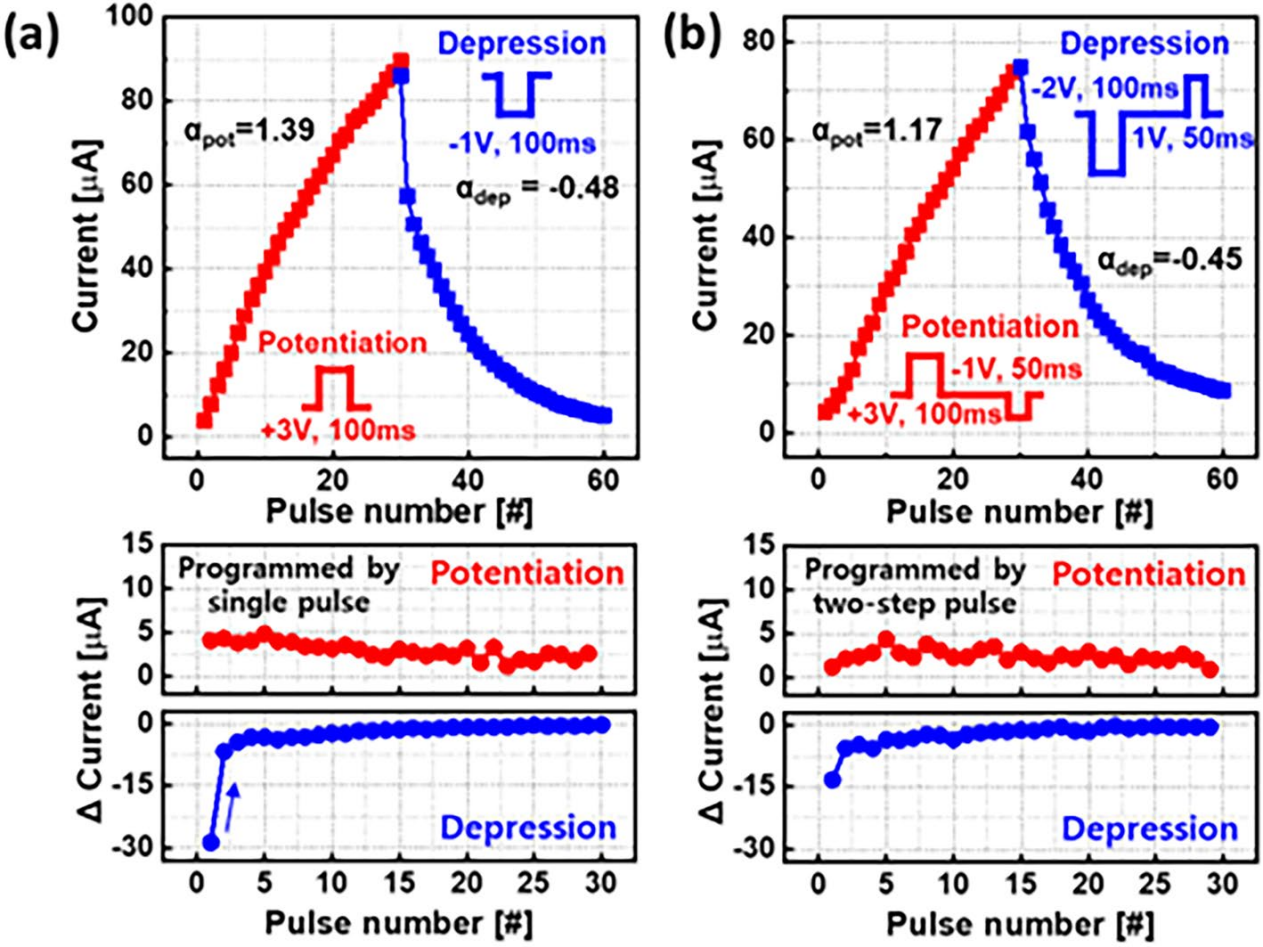
The update curve of I_D_ in the CuO_x_/Al_2_O_3_/HfO_x_ ST programmed by (**a**) conventional single V_G_ pulse method and (**b**) two-step programming pulse scheme. As a result, the degree of the I_D_ per V_G_ pulse became uniform even in depression.

Figure 4a shows the analog synaptic behavior of the CuO_x_/Al_2_O_3_/HfO_x_ ST to be compared with that of the CuO_x_ /HfO_x_ ST shown in Fig. 2d. The insertion of the Al_2_O_3_ layer improves the potentiation and somewhat mitigates the initial current drop in the early depression phase. The degree of the I_D_ change per pulse became relatively constant except for the first pulse during the depression stage, resulting in α_dep_ of − 0.48.

While it should be possible to enhance the device architecture further, we chose to demonstrate the effect of using two gate pulses instead of just one. Essentially, a short pulse of opposite polarity with a width of 50 ms was added to the conventional single V_G_ pulse (see inset to Fig. 4b). Naturally, adding the opposite polarity pulse reduced the dynamic range in Fig. 4b compared to Fig. 4a. However, the linearity was much improved, and almost symmetric synaptic behaviors were obtained. Most notably, the initial depression drop was mitigated in the ST using the CuO_x_/Al_2_O_3_/HfO_x_ stack. The calculated linearity parameters α_pot_ and α_dep_ are 1.17 and − 0.45, respectively.

**Figure 5:**
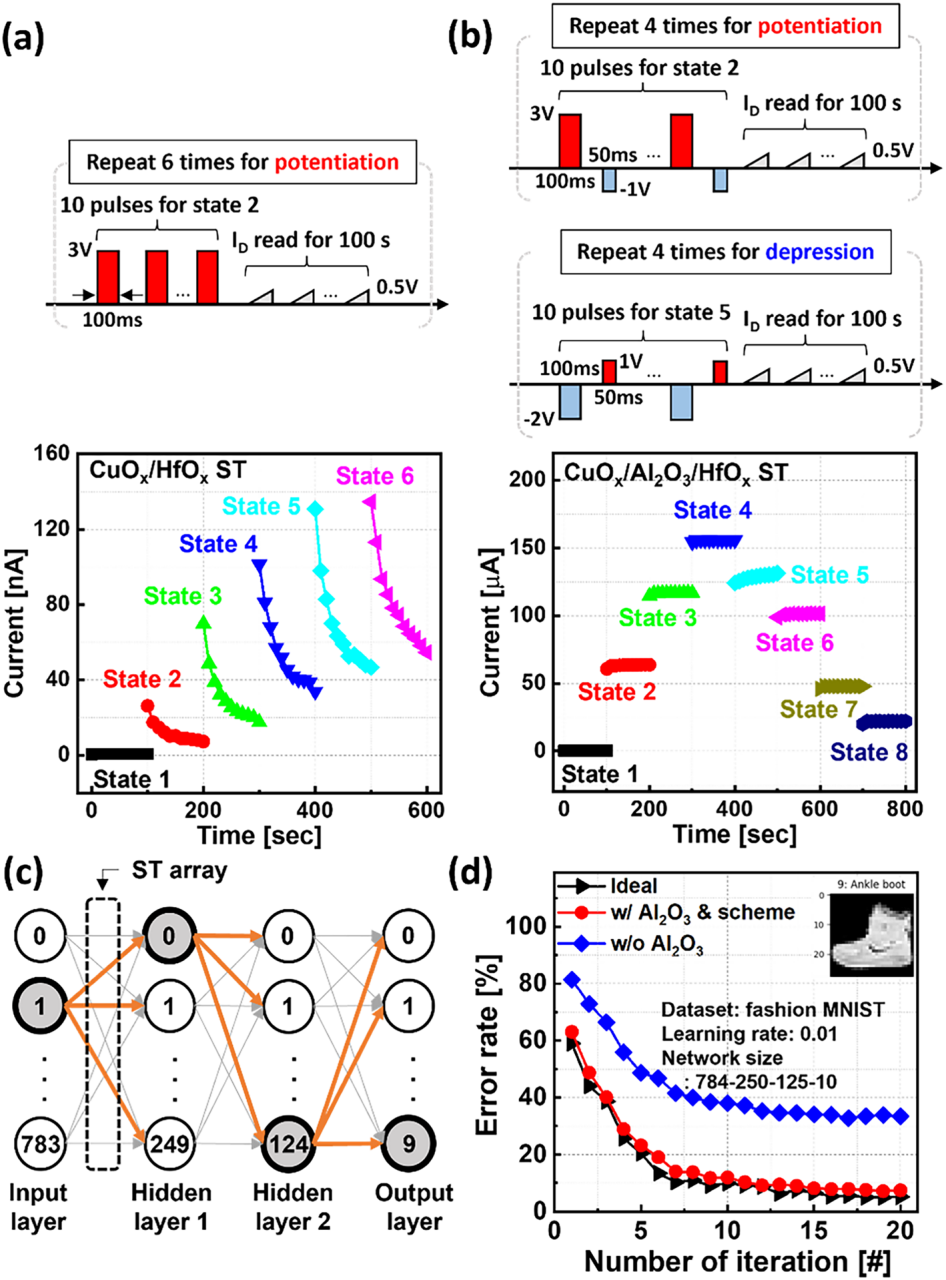
(**a**) Multiple I_D_ states over time in ST with CuO_x_/HfO_x_. (**b**) Reliable I_D_ states for programmed and erased by two-step programming pulse scheme in ST with CuO_x_/Al_2_O_3_/HfO_x_ stack, measured at room temperature for 100 s. (**c**) Schematic of four-layer-based artificial neural network. (**d**) Recognition accuracy results in the case with and without Al_2_O_3_ layer and programming scheme. Near-ideal accuracy was evaluated in ST with CuO_x_/Al_2_O_3_/HfO_x_ programmed under two-step pulse scheme.

To show that the Al_2_O_3_ layer confines the Cu ions to the HfO_x_ layer and acts as partial barrier to Cu ion transport, we tested the state retention during analog switching (Fig. 5). As the top of Fig. 5a shows, the test procedure has a basic block consisting of 10 potentiation pulses followed by a sequence of read pulses for 100 s. This block is then repeated several times. In the context of Fig. 2, we mentioned that the sublinear potentiation is probably associated with poor state retention and fast discharge during potentiation. Figure 5a clearly shows that the CuO_x_/HfO_x_ ST has poor retention. In contrast, the response of the CuO_x_/Al_2_O_3_/HfO_x_ ST (Fig. 5b) shows a stable state-retention allowing us also to test the stability during depression. Namely, the Al_2_O_3_ layer is acting as a barrier confining the Cu ions to the HfO_x_ layer and thus preventing state discharge during read operation.

Finally, we built a multilayer neural network comprising the input, hidden, and output layers based on backpropagation algorithms, as demonstrated in Fig. 5c. The input, first hidden, second hidden, and output layers were composed of 784, 250, 125, and 10 neurons, respectively. The signals were transferred from input neurons to output neurons through synaptic weights, which served as the fabricated STs in this study. The recognition accuracy on the Fashion-MNIST dataset was evaluated using an IBM analog hardware-acceleration simulator kit (AIHWKIT) with a learning rate of 0.01^30^. When highly asymmetric synaptic behavior owing to abrupt I_D_ drop during depression obtained from a CuO_x_/HfO_x_ ST stack was used, low recognition accuracy of approximately 67% was achieved, which corresponds to an error rate of approximately 33% at 20 iterations, as shown in Fig. 5d. On the other hand, the near-ideal recognition accuracy of approximately 93% with significantly lower error rate of 7% was obtained by exploiting a CuO_x_/Al_2_O_3_/HfO_x_ ST.

To conclude, we introduced analog switching based on a structure that avoids the use of WO_x_ channel layer, letting the HfO_x_ take the role of both the electrolyte and the conducting channel. For this, the stoichiometry of the HfO_x_ had to be fine-tuned to support stable Cu ion transport as well as electron transport (probably via trap-assisted tunneling)^31^. A sweet spot in terms of oxygen vacancies was found for the process involving 25:5 Ar:O_2_ sccm flow. However, a 5 nm HfO_x_ that is directly attached to the Cu ion reservoir (CuO_x_) exhibits poor state retention as the Cu ions are easily pulled back into the CuO_x_ layer. This resulted in a sub-linear potentiation response and, more pronouncedly, a 50% drop during the first depression pulse (Fig. 2). To mitigate the facile pullback of Cu ions, we use an ALD process to introduce a relatively dense and stoichiometric Al_2_O_3_ layer between the HfO_x_ and the CuO_x_. EDS studies (Fig. 3) showed that the Al_2_O_3_ layer acts as a barrier confining the Cu ions to the HfO_x_ layer. Consequently, the CuO_x_/Al_2_O_3_/HfO_x_ stack shows improved state retention and a better linear response (Figs. 4,5). Lastly, to test the quality of the CuO_x_/Al_2_O_3_/HfO_x_ ST response, we implemented it to simulate pattern recognition using IBM AIHWKIT, resulting in an error rate as low as 3%.

## Data Availability

The data that support the findings of this study are available from the corresponding author upon reasonable request.

## References

[1] LeCun Y, Bengio Y, Hinton G (2015). Deep learning. Nature.

[2] Mead C (1990). Neuromorphic electronic systems. Proc. IEEE.

[3] Bian J, Cao Z, Zhou P (2021). Neuromorphic computing: Devices, hardware, and system application facilitated by two-dimensional materials. Appl. Phys. Rev..

[4] Wan Q, Sharbati MT, Erickson JR, Du Y, Xiong F (2019). Emerging artificial synaptic devices for neuromorphic computing. Adv. Mater. Technol..

[5] Burr GW, Sebastian A, Vianello E, Waser R, Parkin S (2020). Emerging materials in neuromorphic computing: Guest editorial. APL Mater..

[6] Seo J-S, Brezzo B, Liu Y, Parker BD, Esser SK, Montoye RK, Rajendran B, Tierno JA, Chang L, Modha DS, Friedman DJ (2011). IEEE Custom Integrated Circuits Conference.

[7] Kim M-K, Kim I-J, Lee J-S (2021). Oxide semiconductor-based ferroelectric thin-film transistors for advanced neuromorphic computing. Appl. Phys. Lett..

[8] Lv W, Cai J, Tu H, Zhang L, Li R, Yuan Z, Finocchio G, Li S, Sun X, Bian L, Zhang B, Xiong R, Zeng Z (2022). Stochastic artificial synapses based on nanoscale magnetic tunnel junction for neuromorphic applications. Appl. Phys. Lett..

[9] Burr, G. W. *et al.*.Experimental demonstration and tolerancing of a large-scale neural network (165,000 synapses), using phase-change memory as the synaptic weight element. IEEE International Electron Devices Meeting. 29.5.1-29.5.4 (2014).

[10] Eryilmaz, S. B., Kuzum, D., Jeyasingh, R. G. D., Kim, S., BrightSky, M., Lam, C., Wong, H.-S. P., *IEEE International Electron Devices Meeting*, 25.5.1–25.5.4, (2013).

[11] Bichler O, Suri M, Quelioz D, Vuillaume D, DeSalvo B, Gamrat C (2012). Visual pattern extraction using energy-efficient “2-PCM Synapse” neuromorphic architecture. IEEE Trans. Electron Devices.

[12] Woo J, Yu S (2018). Resistive memory-based analog synapse: The pursuit for linear and symmetric weight update. IEEE Nanotechnol. Mag..

[13] Woo J, Moon K, Song J, Lee S, Kwak M, Park J, Hwang H (2016). Improved synaptic behavior under identical pulses using AlO_x_/HfO_2_ bilayer RRAM array for neuromorphic systems. IEEE Electron Device Lett..

[14] Cuppers F, Menzel S, Bengel C, Hardtdegen A, von Witzleben M, Bottger U, Waser R, Hoffmann-Eifert S (2019). Exploiting the switching dynamics of HfO_2-_based ReRAM devices for reliable analog memristive behaviour. APL Mater..

[15] Woo J, Moon K, Song J, Kwak M, Park J, Hwang H (2016). Optimized programming scheme enabling linear potentiation in filamentary HfO_2_ RRAM synapse for neuromorphic systems. IEEE Trans. Electron Devices.

[16] Zhao M, Gao B, Xi Y, Xu F, Wu H, Qian H (2019). Endurance and retention degradation of intermediate levels in filamentary analog RRAM. J. Electron Devices Soc..

[17] Tang, J. *et al*. ECRAM as Scalable Synaptic Cell for High-Speed, Low-Power Neuromorphic Computing. IEEE International Electron Devices Meeting. 13.1.1-13.1.4 (2018).

[18] Tessler N, Kim N, Kang H, Woo J (2023). Switching mechanisms of CMOS-compatible ECRAM transistors—Electrolyte charging and ion plating. J. Appl. Phys..

[19] Fuller, E. J. *et al*. Li-ion synaptic transistor for low power analog computing. Adv. Mater. **29**(4), 1–8 (2017). 10.1002/adma.201604310 27874238

[20] Kim, S. *et al.* Metal-oxide based, CMOS compatible ECRAM for Deep Learning Accelerator. IEEE International Electron Devices Meeting. 35.7.1-35.7.4 (2019).

[21] Lee C, Choi W, Kwak M, Kim S, Hwang H (2021). Impact of electrolyte density on synaptic characteristics of oxygen-based ionic synaptic transistor. Appl. Phys. Lett..

[22] Yao X, Klyukin K, Lu W, Onen M, Ryu S, Kim D, Emond N, Waluyo I, Hunt A, del Alamo JA, Li J, Yildiz B (2020). Protonic solid-state electrochemical synapse for physical neural networks. Nat. Commun..

[23] Cui J, An F, Qian J, Wu Y, Sloan LL, Pidaparthy S, Zuo J-M, Cao Q (2023). CMOS-compatible electrochemical synaptic transistor arrays for deep learning accelerators. Nat. Electron..

[24] Kang H, Woo J (2021). Cu-ion-actuated three-terminal neuromorphic synaptic devices based on binary metal-oxide electrolyte and channel. Appl. Phys. Lett..

[25] Kang H, Kim HW, Hong ER, Woo J (2022). Analog synaptic behavior of mobile ion source-limited electrochemical RAM using CuO_x_ oxide electrode for deep learning accelerator. Appl. Phys. Lett..

[26] Kim N, Kang H, Kim HW, Hong E, Woo J (2022). Understanding synaptic characteristics of nonvolatile analog redox transistor based on mobile ion-modulated-electrolyte thickness model for neuromorphic applications. Appl. Phys. Lett..

[27] Biesinger MC, Lau LWM, Gerson AR, Smart RSC (2010). Resolving surface chemical states in XPS analysis of first row transition metals, oxides and hydroxides: Sc, Ti, V, Cu and Zn. Appl. Surf. Sci..

[28] Luo X, Li Y, Yang H, Liang Y, He K, Sun W, Lin H-H, Yao S, Lu X, Wan L, Feng Z (2018). Investigation of HfO_2_ thin films on Si by X-ray photoelectron spectroscopy, rutherford backscattering, grazing incidence X-ray diffraction and variable angle spectroscopic ellipsometry. Crystals.

[29] Jang J-W, Park S, Burr GW, Hwang H, Jeong Y-H (2015). Optimization of conductance change in Pr_1-x_Ca_x_MnO_3_-based synaptic devices for neuromorphic systems. IEEE Electron Device Lett..

[30] Rasch, M. J., Moreda, D., Gokmen, T., Gallo, M. L., Carta, F., Goldberg, C., Maghraoui, K. E., Sebastian, A., Narayanan, V. *IEEE International Conference on Artificial Intelligence Circuits and Systems*. 1–4, (2021).

[31] Kumar A, Mondal S, Rao KSRK (2016). Structural, electrical, band alignment and charge trapping analysis of nitrogen-annealed Pt/HfO_2_/p-Si (100) MIS devices. Appl. Phys. A.

